# The Therapeutic Potential of Adenosine Triphosphate as an Immune Modulator in the Treatment of HIV/AIDS: A Combination Approach with HAART

**DOI:** 10.2174/157016211796320289

**Published:** 2011-06

**Authors:** Marc C.E. Wagner

**Affiliations:** 1519 Mansion Place, Pittsburgh, PA 15218, USA

**Keywords:** HIV, ATP, inflammasome, purinergic signaling.

## Abstract

Extracellular adenosine triphosphate (eATP) is a potent molecule that has the capacity to modulate various aspects of cell functions including gene expression. This element of modulation is essential to the role of ATP as a therapeutic agent. The hypothesis presented is that ATP can have an important impact on the treatment of HIV infection. This is supported in part by published research, although a much greater role for ATP is suggested than prior authors ever thought possible. ATP has the ability to enhance the immune system and could thus improve the host’s own defense mechanisms to eradicate the virus-infected cells and restore normal immune function. This could provide effective therapy when used in conjunction with highly active antiretroviral therapies (HAART) to eliminate the latently infected cells. The key lies in applying ATP through the methodology described. This article presents a strategy for using ATP therapeutically along with background evidence to substantiate the importance of using ATP in the treatment of HIV infection.

## OVERVIEW

The effect of the HIV virus on the immune system adversely affects homeostasis. Profound changes occur beyond enumeration of helper and suppressor T cells and viral levels. T regulatory cells (Tregs) heighten this dysfunction through their interactions with dendritic and other immune cells, resulting in suppression of the antigen presentation process. This type of activity points to cytokine, chemokine, and second messenger abnormalities leading to a greater degree of tolerance causing the immune system to operate pathologically. The interaction of eATP with cell surface receptors is well documented in the literature since the observations reported by Dr. Geoffrey Burnstock in 1972[1]. The research initially focused on action potentials within the nervous system and has expanded to modulation of the vascular endothelium and the immune system cells leading to a loss or gain in function [2-4].

**Fig. (1):**
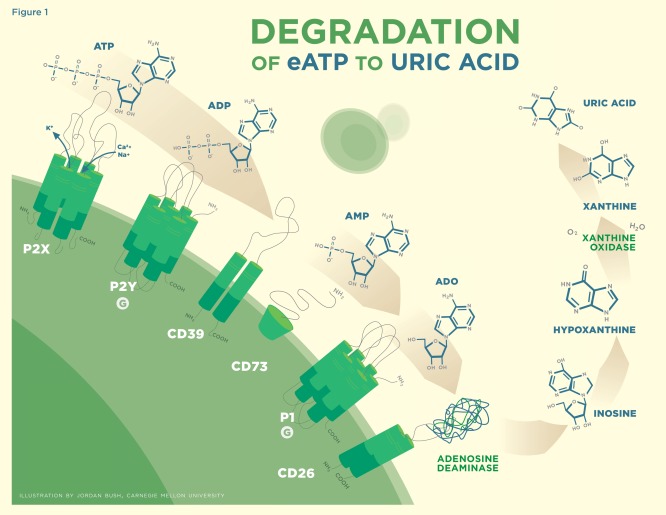
Binding of ATP to the P2X and P2Y series of receptors. ADP preferentially binds to the P2Y series of receptors. CD39 is involved in the catabolism of ATP to ADP to AMP. CD73 is involved in the catabolism of AMP to ADO. ADO preferentially binds to the P1 series of receptors. CD26 in association with adenosine deaminase metabolizes ADO to inosine which is further reduced by xanthine oxidase to uric acid.

ATP is a lipophobic molecule operating as a ligand that binds to receptors on the surface of cells and results in the manipulation of their function. Ectonucleotidase of CD39 catalyzes ATP to adenosine diphosphate (ADP) and further to adenosine monophosphate (AMP), which CD73 (both membrane-bound and soluble forms) catalyzes to the final product of adenosine (ADO), with each metabolite exerting its own specific effects [5]. There is differential expression in both CD39 and CD73 between cell types where Foxp3+ Treg cells contain both, allowing for their participation in the complete hydrolysis of ATP to ADO (Fig. **1**) [6-9]. This is considered a unique feature that defines Treg cell function by subduing an overactive immune response through the generation of ADO from ATP [7, 10]. CD39 expression is identified to a high degree on a subset of Treg cells and is recognized to be a B cell activation marker. CD39 is expressed to a lesser degree on other hemopoetic cells including monocytes, dendritic cells, and natural killer (NK) cells and on activated T cells, erythrocytes, hepatocytes, and endothelial cells.

eATP interacts with the receptor types P2X1–P2X7 ion-gated channels, affecting intracellular sodium, potassium, and calcium levels [11, 12]. The molecule has been shown to stimulate sodium influx into human lymphocytes by 3–12 fold [13]. eATP opens permeablization pores of 900 Da in macrophages and 400 Da in lymphocytes, allowing small molecules to pass through the cell membrane [14]. Intracellular ionized free calcium is a signaling molecule that is kept tightly regulated between 10–200 nM concentrations, and oscillations in these levels result in changes in cytoskeletal protein rearrangements. eATP also binds to the P2Y family of transmembrane proteins embedded in the lipid bilayer [12]. P1 receptors with the subtypes of A(1), A(2A), A(2B), and A(3) are associated specifically with the interaction with ADO as well as with the down-regulation of immune responses [12]. All three of these major receptor pathways and their subtypes are involved in the signaling cascade that assists cells in recognizing and responding to their environment. Activation of the P1 receptor will likely further enhance the actions of eATP by resetting the stage.

eATP indirectly modulates cell function in several ways. One is through the tyrosine kinase receptor as a result of increasing the release of two growth factors: vascular endothelial growth factor (VEGF) and fibroblast growth factor (FGF2), both of which affect growth and differentiation [15-18]. eATP also modulates cell function indirectly through the nitric oxide (NO) pathway. NO is released from activated cells resulting in an increase of intracellular cyclic GMP (cGMP) in target cells. The consequences of these changes are measured in alterations in the expression of cytokines, chemokines, and second-messenger molecules. Signal transduction initiated by eATP after binding to these receptors results in such actions as increasing phospholipase C and D [19-21]. The downstream effect of increasing phospholipase C is the cleaving of phosphatidylinositol 4,5-bisphosphate (PIP2) into inositol triphosphate (IP3) and diacylglycerol (DAG), which in turn act as second messengers coordinating their efforts [22-25]. IP3 will lead to increased release of calcium from intracellular stores reacting with phosphokinase C (PKC). When PKC binds to DAG, it results in the phosphorylation of a myriad of proteins, which either turn on or turn off function.

When it interacts with the P2Y receptor, eATP has been shown to be involved in the regulation of glycogen metabolism through the activation of glycogen phosphorylase [26]. This action catalyzes the most accessible glucose found in glycogen stores, converting it to glucose-1-phosphate and resulting in the release of energy quickly to the cell. The state of the organism determines how the changes will be interpreted. eATP affects mitogen-activated protein kinase (MAPK), which activates transcription factors likely through the binding of VEGF [27]. The activation of VEGF is considered to be a primary factor in angiogenesis, which is a direct effect of a tyrosine kinase autophosphorylation pathway lending to the support and creation of new blood vessels [15, 28]. eATP advances the activation of the kinase cascade *via *ERK1/2 activating transcription factors through the activator protein 1 (AP-1) family, which leads to changes in cytokine and growth-factor expression. This validates the findings in the literature [29]. Cell proliferation is activated through this process. The resulting increase of intracellular free calcium concentration has been found to lead to the phosphorylation of ERK1/2 [29]. Activation of the MAPK and the downstream ERK1/2 pathways is thought to be necessary for NK cell lysis of tumor target cells and is required for NK effector function. Activation of these two pathways is involved in the regulation, mobilization, and distribution of cytoplasmic granules of granzyme B and perforin [30]. NK cell function is compromised by HIV infection. Enhancement of NK cell function brought about by exposure to eATP could have a powerful effect on the elimination of the viral-infected cell [31].

eATP also interacts with the P2Y G coupled protein receptors, eventually leading to increases in the level of intracellular cyclic AMP (cAMP) [32]. This pathway of transduction amplifies responses by the reversible protein phosphorylation of numerous cytosolic enzymes, driving many cellular actions such as the increase in adenosine monophosphate kinase (AMPK). Increasing AMPK leads to the phosphorylation of a number of proteins that act as metabolic masters attenuating glucose uptake and fatty acid metabolism [33]. It is important to note that any agent that would increase AMPK should yield a positive effect on type-2 diabetes [34].

The increase in intracellular cAMP has a direct effect on protein kinase A (PKA), which belongs to the same family as protein kinases B and C (PKB, PKC). It is known in the literature that eATP activates PKA, PKB, and PKC [24, 32, 35]. In addition, the activation of PKA by prostaglandin E2 (PGE2) has been demonstrated to inhibit HIV-1 replication [36]. This effect is proposed to result in the down-regulation of the co-binding receptor CCR5 [37]. Eicosanoids such as PGE2 are increased as a result of HIV infection over controls; this is perceived to be part of the disease process [38].

The culmination of these advanced stages of cellular signaling is a cascade of molecular events yielding a change in cellular responsiveness. ATP binding to cell-surface purinergic receptors is responsible for relaying information from extracellular stimuli to the cell’s core regulatory mechanisms [39, 40]. Activation of PKC has been associated with proliferation, growth, differentiation, apoptosis, and the enhancement of the antigen-mediated T-cell activation. eATP induces the generation of neurotrophic factors such as FGF2, a potent angiogenic molecule that is also involved in tissue repair and the growth of smooth-muscle and hematopoetic cells. FGF2 may well work with VEGF in some of these actions, and CD39 may be associated in these responses. ATP released during injury activates epidermal growth factor receptors that enhance the healing process [41].

Increased NO production acts as a potent vasodilator with an ability to increase intracellular cGMP [20]. NO protects the circulatory systems by decreasing blood pressure and lowering bad cholesterol levels; thus, in sufficient quantities, it improves cardiac function. Madhusoodanan and Murad demonstrated that nanomolar concentrations of NO would increase intracellular cGMP [42]. The real value may be in evaluating the ratios of cAMP to cGMP in subjects as ATP is applied clinically. *In vitro,* there appears to be a point at which increasing the dosage of eATP does not yield an increase in NO release from platelets [43]. This may reflect a saturation effect, a feedback mechanism, a state of equilibrium, or a product of desensitization. Desensitization is a fundamental example in homeostasis brought into play as a cell attempts to reach the basal state, which may be experienced in the clinical application. Desensitization has been identified in *in vitro* experiments as a natural phenomenon that can occur when a cell is exposed to eATP [44]. Endogenous sources of eATP are very small compared with the proposed application of this molecule, which leads to a differential effect exerted on immune system function. Exogenous treatment with ATP can achieve a broader and more substantial degree of change in immune activation, pushing the envelope of transformation.

The complexity of immune system function is rivaled by the complexity in eATP action [45]. The plasticity of the immune system can generate opposing effects –– in some cases inhibiting and in other cases strengthening particular actions. Thus more than one possibility or scenario can result from an action, yielding more than one conclusion.

The final outcome of the hydrolysis of ATP is ADO creating a type of ying-yang effect, which augments cellular responses as ADO down-regulates immune responses [19]. Being stimulatory, inhibitory, and/or bifunctional, eATP will exhibit the same range of potential yielding at times antagonistic roles. As an example, purinergic receptors are important players in both apoptosis and growth. eATP is a key physiologic molecule known to activate purinergic receptors, which are widely expressed among all cell types, triggering a cascade of downstream events with the inducible potential to cause cells of the immune system to alter function [46]. The activation of these receptors has a significant impact on cellular communication, adhesion, motility, proliferation, differentiation, development, death, regeneration, neurotransmission, and gene expression [47]. Increased eATP is currently thought to be associated with pathogenesis. A compelling and convincing argument can be made that eATP is more important in the repair process [15] [48].

ATP initiates a pro-inflammatory response and is involved in the creation of inflammasones, representing a fundamental action that the innate immune system takes against an invading pathogen. ATP-induced autophagy of intracellular mycobacteria may overlap with this mechanism [49] [50] [51]. ADO, the downstream catalytic product of ATP, quiets the immune system. This sounding of the alarm and call to action by eATP, followed by the moderation imposed by ADO, represents a self-regulating system [52] [53]. eATP binding initiates signal transduction and results in transcription, translation, and second-messenger modulation that ultimately invokes a change in immune system action [54]. ATP orchestrates these changes on the HIV-infected immune system by altering how cells interact and react, leading to events such as the maturation of dendritic cells, the regulation of Tregs’ function, and an increased capacity for phagocytic activity. The culmination is a direct result of adjusting the level of expression and secretion of many cytokine, chemokine, and second-messenger molecules.

eATP is emerging as a potent regulator of Treg function [55]. A subset of Treg cells known as Foxp3 + express high levels of CD39, potentially representing the inducible Tregs. The expression level of CD39 has been shown to be increased by cAMP, which results in an improved capacity for ATP to metabolize to AMP [56]. The presence of increased levels of eATP and/or its degradation products ADP, AMP, and ADO, acting through purinergic signaling, has been shown to alter the expression of these important effector molecules: IL-1, IL-2, IL-4, IL-6, IL-8, IL-10, IL-12, IL-18, IL-23, TNF-α, thrombospondin-1, PGE2, prostacyclin, arachidonic acid, FGF2, VEGF, cAMP, cGMP, NO, and intracellular sodium, potassium, and calcium.

Table **1** itemizes the effect of eATP observed on each of these effector molecules and references the relevant research. The changes in these bio-molecules generated by ATP exert a significant effect on cells and on overall physiology because of the common language that cells use to communicate. The pattern of activation will vary by the target cell affected and by the role of each factor within the system as a whole. There exists an intimate relationship between these cellular communication molecules and how a cell functions that ultimately changes the outcome for the disease process in an individual.

There are over 30 trillion cells in the human body, each participating in a collaborative effort to reduce risk and allow for an individual to thrive. Proper communication between cells is essential for homeostasis. Dysfunction is characterized by something going wrong in the conversations between and among cells leading to a disease state. It is postulated here that all the bio-molecules altered by the presence of eATP work synergistically to bring about the improved health of the infected individual through modification of the communication biology.

It has been proposed in the literature that a duality of response that has been observed with eATP is concentration dependent. Low levels of eATP have been shown to inhibit platelet aggregation, while higher levels increase the potential for aggregation *in vitro* [57]. This dose-difference effect may result from the rate-limiting process of ATP catalyzing to AMP, where ADP is more prothrombotic [58]. ADP binding to either the P2Y1 or P2Y12 receptor on platelets leads to fibrinogen-receptor activation and contributes to platelet aggregation. The *in vivo* sources of eATP are associated with apoptosis or necrosis or are released from cells either through exocytosis or by shear force as occurs when red blood cells move through small veinules and arterioles [59]. Activated lymphocytes release ATP by means of exocytosis through the pannexin-1 hemichannel, which acts as an autocrine stimulator and is thought to be crucial for effective activation of T cells, likely through the activation of PKC [2, 60, 61]. eATP might down-regulate Treg cells by up-regulating the secretion of IL-1β by activated macrophages and dendritic cells. The activation of the cysteine protease caspase – 1 converting enzyme is involved in the maturation and secretion of IL-1β and IL-18 through the cleaving of the precursor forms. Upon secretion, IL-1β and IL-18 act as known anti-Treg cytokines shifting immune cells from a Th2 to a Th1 profile [62]. Caspase-1 is involved in the same manner in the processing of IL-23 and IL-33.

Treg cells are recognized as a pivotal component in immunosuppression with responsibility for inducing tolerance and controlling the activity of the immune system. Given what is known about the effects of eATP on cell physiology, one could anticipate in theory that IL-1 and IL-12 released from macrophage or dendritic cells would share the ability to excite naïve T helper cells, enhancing their clonal expansion. One could also anticipate that the ensuing increase in IL-2 release would act in converting cytotoxic T cells into active killer cells and would cause B cells to transform into plasma cells, thus boosting immunoglobulin production specific to HIV antigens [63]. Activated B cells express high levels of CD39 correlating to their function. In combination with the naïve T helper cells, the release of IL-12 from eATP-activated dendritic cells also induces the increased expression of gamma interferon (IFN-γ) as part of the gamma interferon axis that interfaces the innate and adaptive immune systems. The release of IL-18 promotes IFN-γ production by NK cells, which promotes naïve T helper cells into a Th1 profile. The increased secretion of IL-18 exerts a suppressive action on Treg cell function. These responses represent critical countermeasures in the fight against invading pathogens.

Survival depends on the elimination of unnecessary or dangerous cells through more than one mechanism of cell death by apoptosis. This is a conventional process involved in normal tissue and organ maintenance. Diseases arise when this process becomes dysregulated [64]. Increased apoptosis is a hallmark of conditions such as HIV, Alzheimer’s, ischemic damage, and graft versus host disease to mention a few. This process has molecular mechanisms that are normally genetically regulated and kept in balance with Treg cells playing a crucial role.

HIV involves apoptosis of CD4 cells and limits the production of naïve T cells by inhibiting the process of renewal of immune competent cells. The inhibition of T-cell renewal is thought to result from the increased production of IL-7 induced by HIV infection [65]. Spranzi *et al.* evaluated eATP and found that it can trigger apoptosis and programmed cell death in human monocytic leukemia cells and certain tumor cell lines in culture following a rapid increase in intracellular calcium levels [66]. This group of researchers also found that the process of lysis was further enhanced in the presence of gamma interferon. This process was determined to be specific to eATP since the metabolites of ATP were unable to yield the same level of effect on apoptosis. Inhibitors of the calcium-binding protein calmodulin interrupted this process of apoptosis initiated by eATP. Thus calmodulin was linked as a target of change when a cell was treated with eATP. Calmodulin is known to activate over 20 different enzymes as part of its function.

**Fig. (2):**
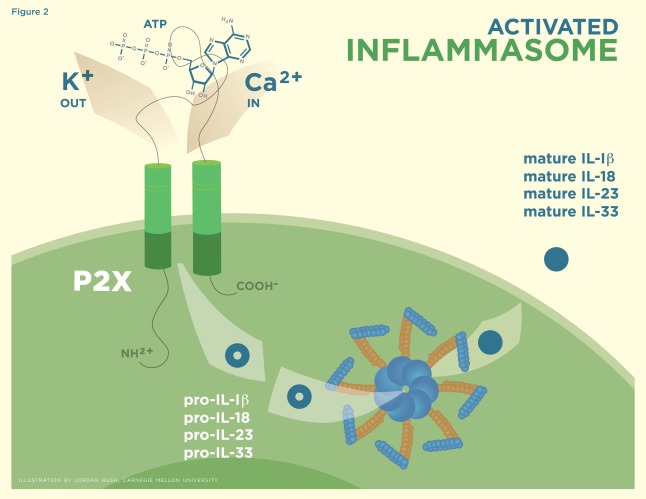
Binding of ATP to the P2X receptor, which facilitates the efflux of K+ ions which causes the formation of the activated inflammasome. The inflammasome is responsible for the maturation of Pro IL-1β, 18, 23, and 33 into their bioactive forms.

Zheng *et al.* further validated that eATP has the capacity to trigger apoptosis or induced programmed cell death in thymocytes, although the mechanism by which this happens has not been clearly identified [67]. Caspase-1 activation occurs when eATP binds to the P2X7 ion-gated channel triggering the process of apoptosis by means of a potent pro-inflammatory response with the assembly of the inflammasome [68-70]. Subsequent stimulation of the P2X7 receptor results in the efflux of potassium, causing the assembly of inactive, nucleotide-binding domain, leucine-rich repeat proteins (NLRP3) with apoptosis-associated speck-like protein containing a CARD (ASC) and pro-caspase-1. The activated inflammasome formed from this assemblage results in the maturation of IL-1β, IL-18, IL-23, and IL-33 into their bioactive forms (Fig. **2**) [71-73]. Endogenous eATP and uric acid can act as danger signals in initiating this type of response [74]. It is interesting to note that these two molecules denote the beginning and the end of the catalytic process. When eATP binds to the P2X7 receptor, intracellular potassium concentration decreases, causing caspase-1 to switch from being a protective agent involved in enhancing cell survival to being the source of a suicide pathway determining cell fate by pyroptosis [75,76]. NF-κB has been shown to be up-regulated upon eATP exposure *in vitro,* which may contribute in the pro-inflammatory apoptosis process [77].

This reaction illustrates an efficient way for pathogens to be cleared by the innate immune system and highlights an important possible role for eATP in helping to eliminate latently infected cells [62, 78-80]. Quiescent cells latently infected with HIV, when activated by eATP, are more likely to undergo this host-beneficial process of inflammation that is defensive in nature and protective to the organism in clearing the infectious pathogen [81]. eATP aids in producing the find-me signal that promotes phagocytic clearance of apoptotic cells. This makes sense given that ATP would be released during the process of apoptosis. A two-part mechanism is involved that enhances the release of IL-8, which then escalates the movement of phagocytic cells toward the source in a sort of search-and-destroy mission [82]. It is a fascinating scenario.

NO is also involved in this process. When it is produced by macrophages *via *inducible nitric oxide synthase (iNOS), NO can induce apoptosis. When it is produced by endothelial cells *via *eNOS, however, NO is involved in regulating vascular function and can inhibit apoptosis. iNOS expressed *via *the immune systems is a defense against pathogens. Upon stimulation with eATP, there is an increase in iNOS messenger ribonucleic acid (mRNA). This is stabilized by MAPK, favoring the production of NO [83]. NO and prostacyclin released by endothelial cells cause circulating platelets to remain inactive, reducing the potential for clot formation. eATP also enhances the production of superoxide, which represents a biologically toxic molecule and is produced by the immune system in an effort to fight invading pathogens [32,50].

eATP binding to the P2X7 receptor has been shown to be involved in lymphocyte trafficking by promoting the shedding of CD23 and leukocyte-selectin (L-Selectin [CD62L]) [84,85]. Binding to P2YR subtypes, eATP can influence the migration and recruitment of immune cells, thus enhancing the immune response [86]. Chemokines, which are altered by eATP, assist in this process by which dendritic cells and T cells meet in lymphoid organs. The CCR5 chemokine receptor is down-regulated by eATP, while the CXCR4 receptor is up-regulated as a function of dendritic cell maturation [87,88]. The up-regulation of CXCR4 is likely due to the increase in intracellular cAMP. To a lesser extent than extracellular uridine triphosphate (eUTP), eATP has demonstrated a down-regulatory effect on CXCR4 [89]. This could have a significant effect on the entry of CCR5, CXCR4, and dual tropic viruses. eATP has demonstrated an ability to modulate selectins derived from endothelial cells (E-selectin), L-selectin, and platelet-selectin (P-selectin) [77]. Integrins are cell surface proteins that play a role in adherence to the extracellular matrix and are involved in transmembrane bi-directional signaling and are also affected by eATP, likely through oscillations in intracellular calcium levels [90]. These events can impact cell growth, survival, proliferation, adherence, and migration.

There are several reviews of the effects of eATP on cancer, which detail the majority of the research on ATP to date [91-93]. Even though this field of study was in its infancy in 1990, it was considered to be so meaningful that the *Annals of the New York Academy of Sciences* published a book at that time about the biological actions of eATP [94]. *Purinergic Approaches in Experimental Therapeutics* published in 1997 further explores this topic [95]. The 2010 publication of *Extracellular ATP and Adenosine as Regulators of Endothelial Cell Function,* which focuses on several decades of research on the impact of eATP and ADO as regulators of endothelial cell function, affirms the continued importance of this area of study.

There is abundant and widely accepted evidence in the literature documenting the cellular modulation capacity of eATP, which is at the core of its therapeutic potential [25, 96-99]. Some controversy exists as to whether the effects resulting from eATP are a benefit and or a liability. The same is true for many bioactive molecules such as NO.

The administration of ATP for the treatment of diseases demands consideration and warrants additional research. The process of developing the hypothesis presented here has taken almost 20 years and continues to evolve. The proposed rationale has been the product of observation, review of the literature, understanding, and a vision of what could be achieved with the use of this molecule. For many years, there was considerable resistance in the scientific community to the idea that eATP was involved in cell communication [100]. Conventional wisdom suggested that ectonucleotidases would quickly convert ATP to ADP, to AMP, and to ADO prior to eliciting a response. Indeed it was thought to be advantageous for cells to control the level of eATP since it has such a significant effect. Although the literature does support that hydrolysis occurs rapidly, it does not occur prior to receptor activation. Each of the metabolites of ATP exerts its specific effects.

Substantial advances have occurred in this field in the past 20 years, redefining and expanding the role of eATP in the modulation of cell function and its action on downstream targets. The time is right to pursue this inquiry to its full potential. As researchers, we need to remain open in our quest for the truth in order to be able to pick up on subtle clues that could lead to a fundamental increase in our understanding. The connection between eATP and the treatment of disease is not immediately obvious. This utility may provide the niche for ATP in the treatment of disease. Some of the evidence published in the literature supports the views presented here, but other evidence contradicts these views. This discrepancy is a function of the complexity of the immune systems’ components. ATP has been studied most often in single isolated system, and the results are subject to interpretation.

## A STRATEGY FOR THE THERAPEUTIC USE OF ATP

It is a well-accepted fact that eATP has broad effects on many cell systems. Following is a vision to apply this molecule in the way that will achieve the greatest results in the treatment of diseases involving the immune system.

It is essential to establish the right testing model. If the testing model is deficient, the answers may be accurate but the conclusions may be incomplete. An example of this with eATP can be seen in the murine model, which lacks the P2Y11 receptor that is coupled to adenylyl cyclase activation [101]. The best model to evaluate this hypothesis is believed to be *in vivo,* which holds all the diversity of cells types and biologically active molecules with which the virus is interacting on a daily basis.

Infection by HIV is a multistage process involving attachment, co-receptor binding, fusion, and entry of viral RNA into the T helper lymphocyte [102]. HIV is a non-living virus on its own, yet it can integrate itself into the human CD4 cells and exploit the human system for the purpose of reproduction, with significant negative implications to the infected host. Treatment with anti-virals has led to the development of resistant strains, demonstrating the virus’s ability to evolve and adapt.

**Fig. (3):**
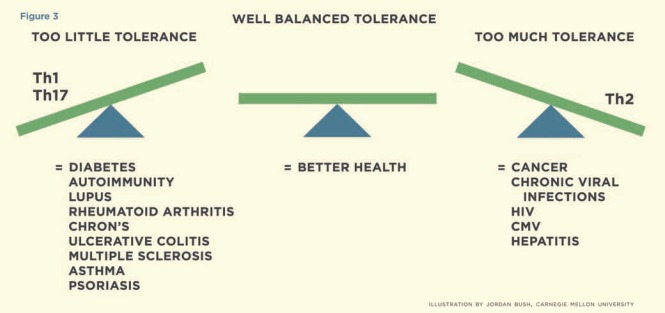
Illustrates conditions, which are associated with too little or too much tolerance. The goal should be to achieve a well-balanced state of tolerance.

The biggest challenge in treating HIV infection is discovering how to eradicate the virus-infected cells. Even powerful HAART therapies have not been able to achieve this result. Latently infected cells remain unrecognized and untouched by the immune systems’ action. ATP has the potential to augment the fundamental function of the innate and adaptive immune systems to target the latently infected cells through activation, apoptosis, and subsequent removal. The co-administration of IFN-γ is expected to accelerate this process. In addition, nicotinamide adenine dinucleotide (NAD) may be added to increase the immune modulation [85]. In essence, ATP could revive normal immune function by altering the immunosuppressive reaction caused by the presence of the HIV virus shifting the balance of tolerance (Fig. **3**) [103].

The human system is an intricate biological machine composed of cells that interact in a sophisticated and eloquent way. Live cell imaging has provided a window by which many cell-to-cell interactions can be seen. Cells respond to environmental cues, and crosstalk is constant. Cytokines can be applied to cells, and their behavior can be observed. In the case of HIV, dendritic cells present the HIV antigens to naïve CD4 T cells for processing and interacting with other immune cells. Alterations in cytokines and second-messenger expression advance the action forward and modulate how cells respond to stimuli and cooperate with one another. The presence of the HIV virus changes these lines of communication over time, leading to a greater level of dysfunction within the host as evidenced by the switch from Th1 to Th2 cytokine expression as the infected individual moves into AIDS. This shift polarizes cells of the immune system towards tolerance. As a single 551-kD molecule, ATP has the ability to affect many of these parameters in an extraordinary manner. Its utility can be exploited in treating patients and ameliorating their condition through the restoration of this communication network.

The force of ATP on HIV is not directly antiviral. For an agent to be directly antiviral, it needs to interrupt the virus life cycle at some key point that can be evaluated in culture testing. ATP does not target specific points of viral production that can be adequately evaluated by culture means. This is further confirmed through the work of Barat *et al.* [88]. The effect of ATP on HIV is more indirect and will be found in its ability to change the microenvironment at the cellular level. The mechanism of these actions will vary depending on how the affected target cells address the commonality that exists in cellular processes being autocrine and/or paracrine in nature [100]. Regardless of how they do that, however, the affected target cells may in turn affect other cells both adjacent and at a distance through the cytokines/chemokines and second-messenger pathways. Some of the most important effects in this application are on the endothelial cells that line the blood vessels, the dendritic cells, and all the hematopoetic cells types [104]. ATP produces a different effect on each of these cell types [103, 105,106].

In 1994, Eliezer Rapaport published a significant article, “Utilization of ATP Administration for the Treatment of Cancer and AIDS”, which focused on cachexia, a common feature of the two diseases [107]. The mechanism of cachexia was believed to be related in large part to an increase in levels of tumor necrosis factor α (TNF-α), which ATP had the ability to decrease. Dr. Rapaport did not recognize that ATP has a much greater role than counteracting physical wasting. The current understanding is that the A_2A_ receptor binding of ADO is responsible for TNF-α inhibition [108].

The hypothesis presented here began with the idea in 1991 of using purine and pyrimidine nucleosides and nucleotides as transport agents for the delivery of elements such as zinc and magnesium. This early work was referred to as NUC-ZM. It was observed that the nucleosides and nucleotides were suitable vehicles for chelated ions. Coordination sites exist where elements have a non-covalent association on the purine and pyrimidine bases [109]. The addition of zinc to double-stranded DNA increases the energy required to dissociate the two strands, thus adding additional stability to the DNA [110]. Others have described these chelates to be rather potent anti-ischemic agents, and observation of their effect confirmed that identification (unpublished observations). Eventually the focus of study moved to ATP as the core molecule with the greatest potential. Although its precursors have a place in science, the most significant value is found in the clinical application of ATP as the therapeutic agent. Oligonucleotides and polynucleotides of various lengths could also be expected to share some of the same pathways of induction if their sequence contains purines. Chemically modified purine nucleotides tri-, di-, and mono-phosphates can be utilized that may be less susceptible to degradation. However, ATP disodium is a safer form to use at least initially given its known safety profile.

The Drug Development and Clinical Sciences Branch of the National Institute of Allergy and Infectious Diseases provides a program to assist scientists in the evaluation of potential therapeutic agents for HIV. Dr. Steven Turk was approached in 1997 to assist in this evaluation by conducting testing on ATP along with adenine, ADO, and AMP in the established T-cell line CEM-SS infected with HIV-1RF or other cytolytic variants of HIV-1 or HIV-2. Drug efficacy and cytotoxicity were determined by the metabolic reduction of tetrazolium salt XTT. The purpose was to determine the antiviral drug activity against the cytolytic variants of HIV-1RF. The results suggested no antiviral efficacy for adenine or ADO but some selective activity for ATP; the results for AMP were questionable because of solubility issues. The results were not impressive enough to warrant additional work on ATP or its precursors as a single-drug therapy. These were unpublished results. ATP is not postulated to have a direct antiviral effect. The hypothesis is centered on ATP’s immunomodulatory importance. Every cell in the immune system experiences its effects.

Bone marrow progenitor cells give rise to three distinct types of dendritic cells: langerhans, interstitial, and plasmacytoid, all of which possess an inherent adjuvant property [111]. These cells supply a link between the adaptive and innate immune processes, and are potent activators of both memory and naïve T cells. Altering the function of these cells can have an effect on inflammatory conditions such as the autoimmune disorders HIV/AIDS, Crohn’s disease, and ulcerative colitis. eATP influences dendritic cell maturation as part of its effect in up-regulating costimulatory and adhesion molecules of CD54, CD80, CD83, and CD86 [112,113]. Maturation initiated by eATP has been shown to coordinate the down-regulation of inflammatory chemokine receptors CCR5 and CXCR4 on dendritic cells [114]. This reduces viral transmission to T helper cells. In chronic HIV infection, the level of CCR5 is up-regulated, which has a significant implication on the earliest point of HIV viral entry and disease persistence. Strains of HIV that are macrophage-tropic are not able to induce syncytia. There is evidence in the literature that eATP in culture affects the antigen presentation process between dendritic cells and CD4 cells, yielding a non-direct mechanism of anti-viral activity by increasing lysosomal degradation and decreasing the transfer of HIV to CD4+ lymphocytes [115]. Once again, changes in the expression of CCR5 are implicated.

Rizzo *et al.* made the correlation between eATP and the immune response to inflammation that is dependent on time, and dose and possesses pro-inflammatory and anti-inflammatory potential [116]. eATP has the ability to enhance immune action through the rapid release of pro-inflammatory cytokines that favor the killing of pathogens. In addition, immune tolerance may be altered through thrombospondin-1, which is released in response to increased levels of eATP [112, 115, 117]. Crombie studied the anti-HIV effect of thrombospondin-1 and found that a 10-fold increase in thrombospondin-1 in saliva was associated with decreasing HIV infectivity orally [117]. Taraboletti identified that thrombospondin-1 inhibits Kaposi’s sarcoma in HIV positive patients [118]. Kumar reviewed for the role of ADO as an endogenous modulator of the innate immune system with a therapeutic potential [119]. As discussed earlier, the catalytic process of ATP eventually leads to ADO.

To date, however, these issues have most often been studied in a one-dimensional culture environment. It is the contention here that the best testing model with a much broader dosage schedule is the more diverse system of an infected person. A human being has all the cell types that can be either directly or indirectly affected by increased levels of eATP. These cells and their actions work in concert with each other-not alone.

Cytokine expression can alter viral activity. Elevated expression of TNF-α increases HIV viral replication [120] [121]. Decreasing a number of the pro-inflammatory cytokines such as TNF-α, IL-4, and IL-6 along with increasing IL-10 can yield an antiviral effect. NO has been shown to be increased in patients with HIV-1 infection [122]. The biological responses observed after an individual becomes chronically infected with HIV can be seen as appropriate steps taken by the immune system in response to the viral infection, yet they fall short of being successful in eradicating the pathogen. This insufficient response is not beneficial overall with respect to the health of the infected individual, especially over time. It leaves the host ill equipped to respond to secondary infections as the immune system deteriorates.

The limited success of ATP in the clinical arena to date may be in part due to the lack of patentability other than use and to the fact that the most effective route of administration is by intravenous infusion. ATP can also be administered by intramuscular injection and orally, although it will likely require a much higher dosage when given intramuscularly or orally compared to intravenously. The application of ATP should not be overlooked since it can have a significant long-term impact on the treatment of diseases related to abnormal tolerance.

ATP has been applied in the clinical setting in two completed trials for the treatment of cachexia in advanced cancer (stage IIIB and IV non-small cell lung cancer) [123-125]. The focus of these two studies was to treat cachexia and improve quality of life performance scores in the patient population. The studies set out to demonstrate a positive effect on these two parameters, and they achieved that. The results did not demonstrate a decrease in tumor size or a notable increase in survival. Widespread apoptosis was not evidenced in the clinical trials as one might expect from the pre-clinical laboratory data. It may be that the results were stunted because of the choice of patients along with the dosage and the duration of treatment. This may have been the best population to demonstrate the anti-cachectic effect and improvement of Karnofsky scores but not the best given the other levels of dysfunction present. Nevertheless, these studies did demonstrate that ATP could be administered safely to patients. This is a very important step that now makes the application of ATP in the treatment of HIV more plausible.

Here is a single ancient molecule with vast potential: it is ubiquitous and can be used as a therapeutic agent. ATP offers an intriguing promise in the treatment of diseases. It will lessen the toxicity of chemotherapeutics and may best be applied as an adjuvant therapy just as proposed here for HIV [126]. ATP will work well in combination with other therapeutics for diseases such as cancer and HIV/AIDS yielding a one, two, three punch.

To ensure clinical success in the application of ATP, the dosage and duration of treatment should be tailored to meet the specific clinical needs of each patient. That will correlate with the greatest possible outcome. To date, a standard dose has been applied clinically to cancer patients: 50 micrograms per kilogram per minute over an 8-12 hour infusion in phase I and up to 96 hours in phase II. Researchers and clinicians are encouraged to think beyond the standard dose and schedule. Hitting the immune system with very high doses of ATP may not be necessary to achieve the desired outcome. A careful massaging may well be more desirable; an approach looking for the maximum efficacious dose may prove more effective as small changes can yield a dramatic impact. There can be considerable advantage in personalizing the dose and schedule to an individual’s clinical needs. The recommended way to do this is to establish disease-specific markers or repair markers for a given condition. In patients with HIV, the parameters to observe include CD4, CD8, quantitative proviral DNA, plasma viral RNA by PCR, neopterine, B2-microglobulin, IL-6, D-dimer, soluble IL-2, CD39 and CD73 expression, C-reactive protein, uric acid, and adenosine deaminase. Other disease-specific markers may be appropriate and should be observed to evaluate the patient’s response to treatment, including any disease- or dysfunction-defining marker that is abnormal at baseline.

Start at 10 micrograms per kilogram per minute and increase or decrease the dosage as needed to achieve a maximum tolerable or maximum efficacious dose. If the patient experiences significant adverse side effects at the proposed dosage, discontinue therapy until any side effect abates and start again at half the original dosage followed by stepwise titration in dosage to reach the maximum tolerable or maximum efficacious dose. The maximum tolerable dose is defined as the highest amount that can be given without complication. The maximum efficacious dose is the point at which disease-specific markers are restored to normal.

Recent clinical trials of ATP did report adverse events in participants at the standard dose and schedule. The most common were chest pain and dyspnea, which resolved within minutes when ATP was discontinued. Another common complication was hyperuricemia, which was treated in study patients with allopurinol given along with ATP. Adenosine deaminase enzyme is essential in the catabolism of adenosine into 2’deoxyinosine and inosine, which is further catabolized into uric acid, a waste product at physiological pH. Uric acid exists as urate that is 30% excreted by the gastrointestinal tract and 70% *via *renal excretions involving filtration, secretion, and post-secretory reabsorption at 6–10%. Normal purine nucleotide metabolism results in the production of uric acid leading to the hyperuricemia experienced in the clinical application of ATP; this is consistent with the known catalytic pathway for ATP [127-130]. Allopurinol inhibits the enzyme hypoxanthine oxidase and prevents the production of uric acid, thus favoring the salvage pathway making more nucleotides. The infusion of ATP should be continued until normal function is restored as assessed by markers.

The goal should be to revive normal cell function through a more structured application of ATP *in vivo*. One dose fits most will be less effective than tailoring the dosage and duration of treatment to meets the needs of the specific individual. It would be reasonable to expect that the level of response seen when a target cell is experiencing eATP would be concentration- and time-dependent, while prolonged stimulation by eATP may temporarily inactivate the receptors through a negative feedback loop. The response will likely vary due to factors such as the level of cell maturation, receptor expression and sensitivity, enzyme activity, and the degree of dysfunction present in the target cells at the time of treatment [131]. Even though this point may seem obvious, there is no evidence in the literature that anyone has attempted to apply ATP in the manner proposed here.

This methodology should be able to be applied in other diseases in an attempt to revive normal cell function by restoring the balance in tolerance. Conditions that could feasible be treated successfully following this methodology include those known to exhibit decreased levels of eATP such as type-2 diabetes, cystic fibrosis, and pulmonary hypertension as well as conditions involving a misdirected immune system such as type-1 diabetes, psoriasis, hepatitis A, B, and C, herpes, CMV, Epstein-Barr viral infections, tuberculosis, rheumatoid arthritis, Crohn’s disease, ulcerative colitis, asthma, and multiple sclerosis [132]. This mode of treatment will be useful in addressing the intersection of immune system activation, inflammation, and the process of chronic illness. There exists a strong connection among phagocytes, complement system, cytokines, and the coagulation systems, all of which are modulated by the presence of increased amounts of eATP. This is plausible given the array of effects ATP can elicit on these targets and its downstream effects. ATP alone can affect the immune system unlike any other single molecule. Once more is known about how to harness its beneficial properties, we may be able to substantially alter disease processes with the use of ATP [133].

The foundation for this hypothesis rests on the work of many scientists such as Dr. Geoffrey Burnstock, Dr. Irshad H. Chaudry, Dr. Pieter C. Dagnelie, Dr. George Dubyak, Dr. Charles M. Haskell, and Dr. Eliezer Rapaport whose contributions built the framework and laid the initial groundwork detailing the various pathways altered by eATP. Their research has answered many questions, but many more need to be addressed. Knowledge constantly evolves, and the proposal put forth in this article represents another step in this process of understanding. Even though this hypothesis is not yet proven as fact, the basic science is already in place. Its benefits will not be fully appreciated until ATP is applied clinically and given proper scientific evaluation in an effort to explore the potential and limitations of this methodology.

## Figures and Tables

**Table 1:** Modulation Effects of Extracellular ATP and Its Metabolites (Direct and Indirect on Cytokine and Second Messenger Expression

Analyte	Action	Source
IL-1	Increased	[19, 60, 62, 134, 135]
IL-1b	Increased	[19, 60, 68, 70, 71, 74, 134, 136-139]
IL-2	Increased Decreased	[48, 63, 140-142]
IL-4	Increased Decreased	[108, 143]
IL-6	Increased Decreased	[62, 108, 138, 144-148]
IL-8	Increased	[19, 82, 149]
IL10	Increased Decreased	[83, 103, 139, 142, 150-153]
IL12	Increased Decreased	[19, 108, 112, 114, 139, 154, 155]
IL-18	Increased	[68, 70, 71]
IL-23	Increased	[19, 137]
TNF-a	Increased Decreased	[19, 136, 144, 150] [108, 136-140, 151, 155]
Interferon γ	Increased	[63, 66, 136]
Thrombospondin 1	Increased	[112, 115]
Prostaglandin E2	Increased	[35, 151, 156]
Prostacyclin	Increased	[23, 157, 158]
Indoleamine 2-3 dioxygenase	Increased	[112, 115]
Intracellular Sodium	Increased	[13]
Intracellular Potassium	Increased Decreased	[68, 71]
Intracellular Calcium	Increased Decreased	[14, 48, 70, 136, 137, 146, 157, 159-163]
Cyclic AMP	Increased	[108, 114, 142, 154, 164, 165]
Cyclic GMP	Increased	[20, 137]
Nitric Oxide	Increased	[20, 43, 74, 137, 150, 157, 158]
Prostaglandin D2	Increased	[23, 164]
Thromboxane B2	Increased	[166]
Arachidonic Acid	Increased	[23, 79, 82]
